# Exploring Attitudes and Obstacles Around Digital Public Health Tools: Insights From a Statewide Cross-Sectional Survey on Washington’s Vaccine Verification System

**DOI:** 10.2196/66550

**Published:** 2025-10-03

**Authors:** Andrea R Molino, Debra Revere, Rebecca A Hills, Adam S Elder, Laura M West, Bryant T Karras, Chris Baumgartner, Janet G Baseman

**Affiliations:** 1 Department of Epidemiology School of Public Health University of Washington Seattle, WA United States; 2 Department of Health Systems and Population Health School of Public Health University of Washington Seattle, WA United States; 3 Department of Health Washington State Tumwater, WA United States

**Keywords:** public health informatics, user survey, evaluation, public health practice, adoption, vaccine, attitudes, beliefs, trust, digital health technology

## Abstract

**Background:**

Development and use of digital public health tools surged during the COVID-19 pandemic. Among these tools, vaccine verification systems emerged as alternatives to paper vaccine records, aiming to help limit the spread of disease. In November 2021, the Washington State Department of Health launched “WA Verify,” a QR code–based vaccine verification system built on the SMART Health Card framework, providing residents with a convenient way to store and share proof of vaccination digitally. However, WA Verify was developed and deployed before assessments and public input regarding potential adoption challenges—such as concerns about privacy, surveillance, data sharing, trust in the technology, and the managing organizations—could be completed.

**Objective:**

This analysis used statewide survey data from Washington to identify and characterize barriers and facilitators to the adoption of WA Verify, and to understand how factors such as data privacy, security, attitudes toward public health policies and communication, and technological proficiency may influence acceptance and uptake of digital public health tools.

**Methods:**

A cross-sectional statewide survey was distributed between September 2022 and January 2023 to a random sample of 5000 Washington households. Respondents were categorized into 3 groups based on their responses indicating WA Verify “users,” “potential users,” or “unlikely users.” Comparisons were made between groups regarding experiences with and opinions on COVID-19 vaccine and test verification, public health policies, communication, digital tools, technological proficiency, sociodemographic characteristics, and health history. Poststratification weights were applied to reduce nonresponse bias.

**Results:**

Of the 1401 respondents, 359 (25.6% unweighted, 25.8% weighted) were users, 662 (47.3% unweighted, 49.8% weighted) were potential users, and 380 (27.1% unweighted, 24.4% weighted) were unlikely users. All percentages reported are based on weighted data. Compared with users and potential users, unlikely users were more likely to oppose policies requiring proof of COVID-19 vaccination or negative test results (users: 6.0%, potential users: 13.6%, unlikely users: 65.9%). Unlikely users were more likely to cite concerns about personal health data security and phone hacking or tracking, though these concerns were also notable among potential users and users. Users and potential users were more likely to perceive a digital vaccine verification system as convenient (users: 96.5%, potential users: 92.3%, unlikely users: 38.1%) and indicated openness to receiving relevant information from a range of sources. Unlikely users were more likely to report not owning a smartphone and demonstrated lower technological proficiency (users: 12.3%, potential users: 15.9%, unlikely users: 32.3%), indicating a technological divide between groups.

**Conclusions:**

While nearly three-quarters of respondents had either already adopted or were willing to adopt a tool like WA Verify, concerns about data security, lower technological proficiency, and distrust of public health characterized those least likely to adopt such tools. Identifying barriers to adoption among “unlikely users” is essential for developing effective communication strategies—such as targeted marketing and community engagement—to improve adoption and ensure equitable access to public health technologies.

## Introduction

The use of digital health tools has surged in recent years, both globally and within the United States. The COVID-19 pandemic acted as a catalyst, spurring the development and subsequent adoption of such tools. This prompted health care systems and public health entities to rapidly and broadly implement technologies such as telemedicine, Bluetooth-enabled contact tracing apps, and digital syndromic surveillance to enhance patient care and protect population health [1-5]. The rollout of COVID-19 vaccines from late 2020 to early 2021 further accelerated this process, as public health agencies, theaters, museums, gyms, workplaces, and other venues sought ways to verify vaccine status, comply with local guidelines, and limit the spread of disease.

**Figure 1:**
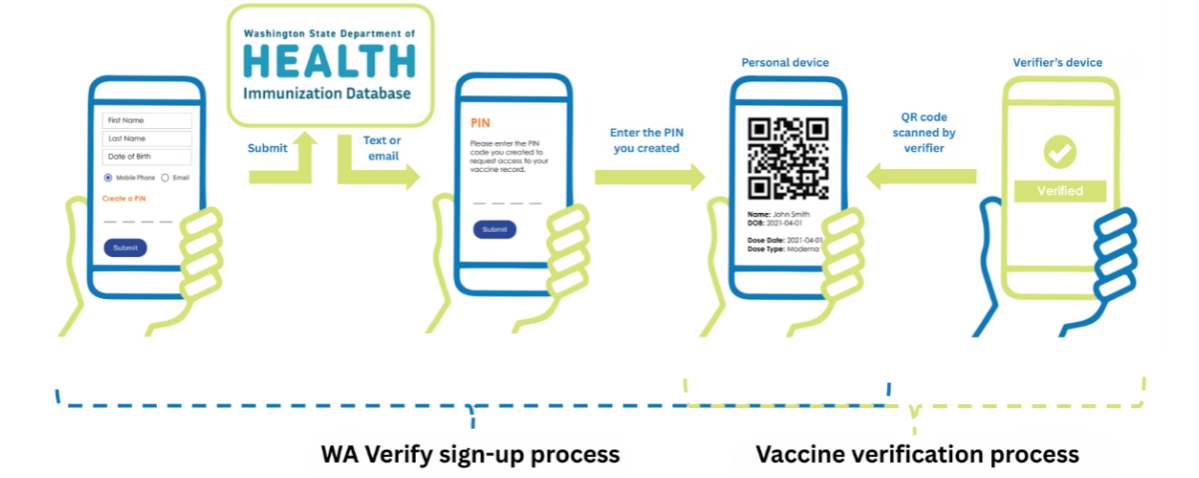
Infographic of WA Verify signup and COVID-19 vaccine verification process.

In Washington (WA) State, public health authorities developed and rolled out “WA Verify,” a digital COVID-19 vaccine verification system based on the SMART Health Card framework [6]. Introduced by the WA State Department of Health (WA DOH) in November 2021, WA Verify was a free and widely used tool that allowed anyone vaccinated for COVID-19 in WA State to easily share proof of vaccination on a mobile device through on-screen text and a QR code, offering residents a convenient, paperless way to keep track of their vaccines [7-10]. Figure 1 shows the WA Verify signup and verification process. A user begins by visiting the WA Verify website, entering personal information, and confirming their identity. They are then granted access to their SMART Health Card and QR code, which can be saved to their Apple (Apple Inc) or Google (Google LLC/Alphabet Inc) Wallet. To update the SMART Health Card with new boosters, the process can be repeated, and a new card will be saved. This process could be repeated for each booster. When entering a space that required proof of vaccination, such as a restaurant or concert venue, a vaccine verifier could scan the QR code, providing the necessary documentation without the need for a paper vaccine card. The WA DOH estimates that, over the course of the program, users sent more than 1.8 million COVID-19 vaccine verification requests to the State’s immunization database.

Because of the urgency of the pandemic, public health tools such as digital vaccine verification systems were quickly implemented, leaving little time to gauge public perception through community outreach. Rapid advancements in health technologies, while often full of promise, can present challenges for widespread public adoption. Consumer health information technologies are not always embraced, even when evidence shows they can benefit individual or population health [11]. Reasons for public nonacceptance of or disinterest in health technologies may include insufficient training, lower levels of “tech savviness,” lack of trust, or low technology self-efficacy [12]. In addition, data security and privacy issues have become increasingly important for users of health technologies, with such concerns exacerbated during the COVID-19 pandemic [13,14]. Apprehensions about the security of health data can hinder the adoption of health-based technologies, undermining the effectiveness of these tools.

Given past challenges to acceptance and the lack of formative work before system rollout, a structured approach to understanding the factors influencing user acceptance is essential to inform this work. The Technology Acceptance Model (TAM) provides such a framework for understanding the adoption of emerging health-focused technologies. TAM proposes that perceived usefulness (the belief that using a given technology can improve the ability to perform a task) and perceived ease of use (the belief that the technology is easy to use) determine attitudes toward technology adoption [15]. While these 2 beliefs can be measured independently, TAM posits that perceived ease of use influences perceived usefulness. Further, TAM proposes that external variables or antecedents—such as social influence, design features, and flexibility—affect a user’s perceived usefulness and perceived ease of use. These beliefs then shape attitudes, influence behavioral intention, and ultimately determine actual use of the technology. In summary, external variables influence perceived usefulness and ease of use, which together drive an individual’s attitudes toward, intention to use, and actual use of a given technology.

While early TAM research in health care technology focused on occupational users such as physicians, nurses, and hospital or clinic personnel [16,17], the literature examining consumer health technology use through the lens of TAM is growing and has been shown to be a valid model for describing patient behavioral intentions [18]. In a recent study examining perceptions of telehealth among an underserved population in rural Michigan, perceived ease of use was found to be a stronger predictor of telemedicine use than perceived usefulness. However, telehealth nonusers were more likely to believe that better medical care would be provided in person and that providers would be less caring via telemedicine [19]. Another recent study, which operationalized social determinants of health as external factors that may predict perceived ease of use and perceived usefulness, found that economic stability and neighborhood environment were predictors of telehealth perceived ease of use during the COVID-19 pandemic. This finding underscores the need to consider how social factors may drive disparities in telehealth adoption [20]. Further related to pandemic health technology usage, perceived usefulness and perceived ease of use have both been shown to be significantly associated with the intention to use COVID-19 smartphone-based exposure notification apps [21], likely motivating individuals to engage in protective behaviors [22]. Additionally, in this age of big data, privacy and security have been recognized as important exogenous factors in TAM, with their impact on technology acceptance increasingly considered, an issue that may be heightened in the case of personal health data [23,24]. As technology becomes increasingly integral to public health systems—and with the pandemic accelerating this shift and changing how people interact with these tools—it is essential to understand public perceptions of digital health technologies, such as vaccine verification systems. Identifying and addressing concerns, misconceptions, and barriers are crucial for broad adoption and acceptance [25,26].

TAM provided a theoretical framework and guided the selection of factors of interest for understanding Verifiable Clinical Information (VCI) tool acceptance and use in this analysis [15]. Specifically, the concept of behavioral intention to use technology informed the categorization of our study population, while the concepts of external variables, perceived ease of use, perceived usefulness, and attitudes toward technology informed the variables selected for comparison. This study aimed to use statewide survey data to identify facilitators and barriers to the adoption of a VCI tool and to understand how factors such as data privacy, security, attitudes toward public health policies and communication, and technological proficiency may influence acceptance and uptake of digital public health tools. The results can help inform public health strategies for system design, rollout, and targeted communication to improve adoption and public trust as the WA DOH develops and implements novel tools to protect population health.

## Methods

### WA Verify Statewide Survey Creation, Distribution, and Data Collection

Data for this analysis come from the WA Verify Statewide Survey, a large multimodal survey distributed to a simple random sample of 5000 residential households across WA State. Addresses were obtained from Marketing Systems Group, and the sample was drawn from an address-based sampling frame using the US Postal Service’s Delivery Sequence File, which includes all known residential household addresses in the state [27]. The goal of the 32-question survey was to better understand the public’s perceptions of the benefits, desired features, and barriers to the acceptance and use of VCI systems such as WA Verify. Survey questions addressed the following topics: technology literacy and access, COVID-19 vaccination verification experience, familiarity with WA Verify, reasons for using or not using the tool, willingness to adopt an electronic vaccine record, opinions on public health policies and digital technologies, and sociodemographic characteristics. To create this survey, we reviewed Knowledge, Attitudes, and Practice surveys and Pew Research Center surveys related to technology use, incorporated demographic questions primarily aligned with United States Census Bureau data, and included questions on behaviors and factors influencing adoption, guided by TAM. The survey was piloted, and feedback was solicited from academic colleagues, students, WA residents, and survey design experts [28].

**Figure 2:**
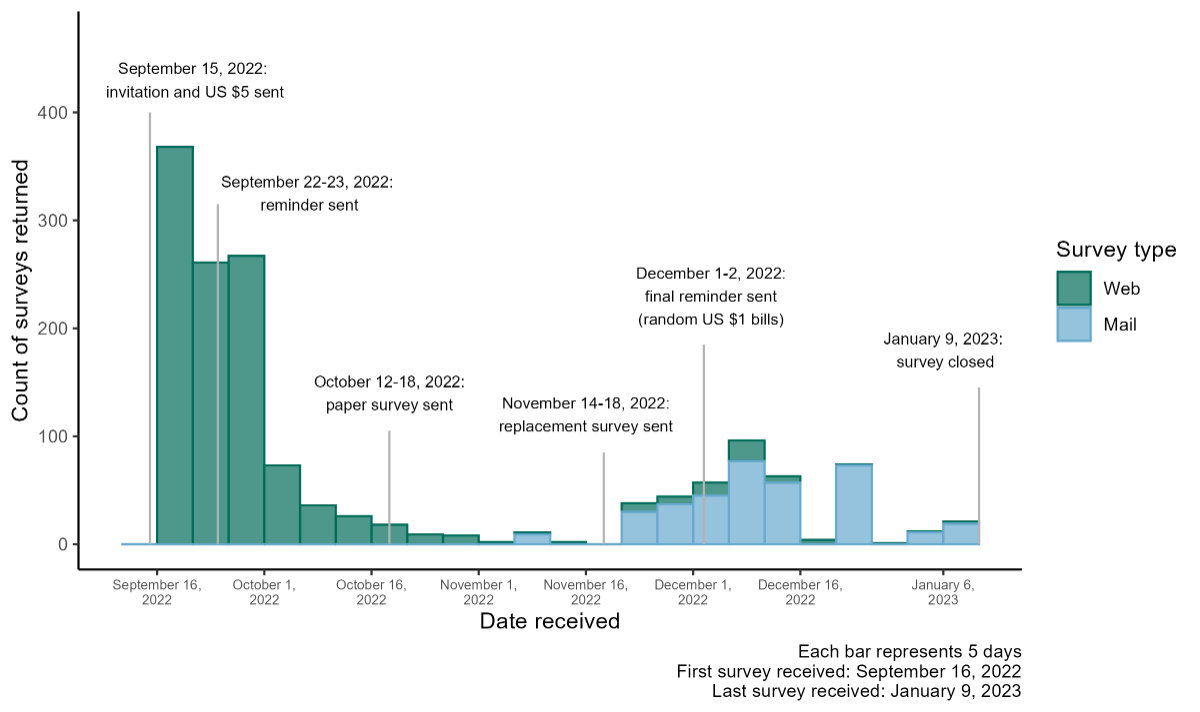
Distribution of web and mail survey responses over time, and relevant survey milestones for the entire survey population (N=1491).

On September 15, 2022, initial invitations were mailed to the 5000 sampled households along with a US $5 incentive. The invitations included instructions for completing the survey online via a QR code or URL. Reminders in English and Spanish, along with a paper version of the survey, were subsequently mailed to nonrespondents in mid-October. A final reminder letter, including a link to the online survey, was sent to nonrespondents on December 1, 2022, with a US $1 incentive included for a randomly selected 900 out of 3422 (26.30%). The survey closed on January 9, 2023, after nearly 4 months of data collection. Figure 2 shows the relevant milestones and the distribution of survey completions over time. The survey was administered through the Social and Economic Sciences Research Center at Washington State University. The full survey instrument is provided in Multimedia Appendix 1.

### WA Verify User Status

**Figure 3:**
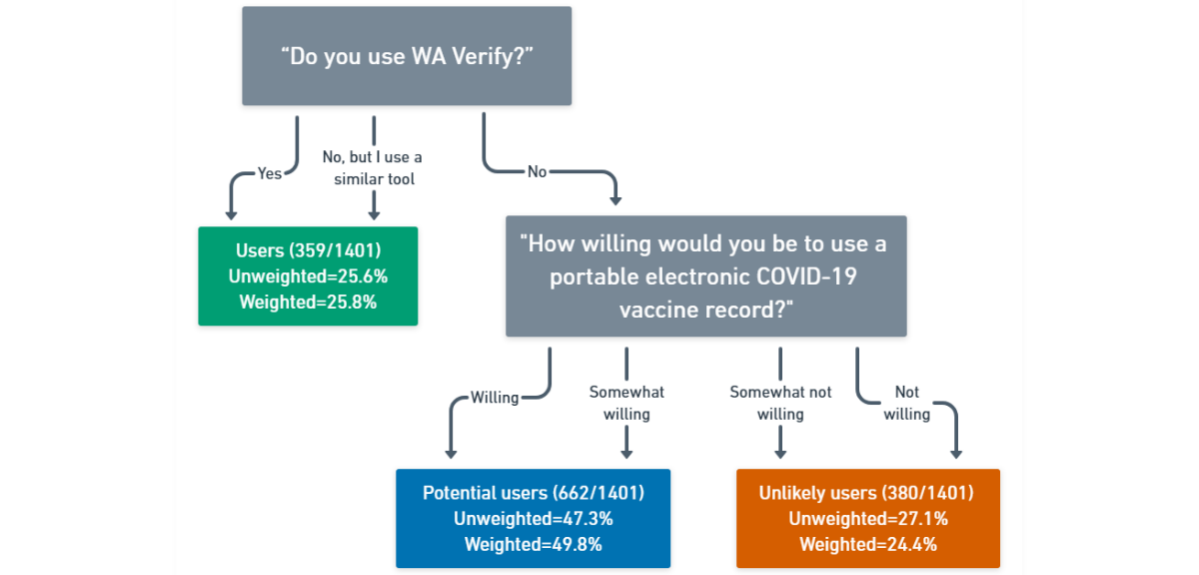
Distribution of user status variable for the analytic sample (N=1401); of these, 90 (6.42%) respondents were excluded from this analysis, as they did not respond to questions required for user status classification. Unweighted sample size and both unweighted and weighted proportions are presented.

The variable of interest in this analysis is “User Status,” which describes an individual’s likelihood or willingness to engage with an electronic tool for vaccine verification, such as WA Verify. This variable was created by categorizing respondents into 3 groups based on their responses to 2 questions. The first asked, “Do you use WA Verify?” with response options of “yes,” “no,” and “no, but I use another, similar tool.” If a respondent reported that they did not use WA Verify or a similar tool, they were asked, “How willing would you be to use a portable electronic COVID-19 vaccine record?” with response options of “willing,” “somewhat willing,” “somewhat not willing,” and “not willing.” *Users* were those who reported using WA Verify or a similar tool; *potential users* were those who did not use WA Verify but were willing to use a portable electronic COVID-19 vaccine record; and *unlikely users* were those who did not use WA Verify and were unwilling to use such a system. A flowchart of these classifications is shown in Figure 3. Survey respondents who did not answer 1 or both of these questions were excluded from the analysis.

### Feelings Toward Policies, Reasons to Use or Not Use WA Verify, WA Verify Information Communication Preferences, and Tech Readiness

The distribution of support for COVID-19 protection policies, reasons for potential use or nonuse of a tool like WA Verify, communication preferences regarding WA Verify and similar information, and “tech readiness” were compared across user status groups. Survey respondents were asked how they felt about policies requiring proof of vaccination or a negative COVID-19 test to enter spaces that might result in disease spread, with response options of “strongly support,” “support,” “oppose,” and “strongly oppose.” Respondents were also asked why they might choose to use a portable electronic COVID-19 vaccine record like WA Verify, with 12 possible reasons provided, and were instructed to select all that applied. This analysis focused on 4 of these reasons—“Having my vaccination card on my phone is convenient,” “Having this tool on my phone would make checking in and accessing health care facilities and my providers quicker,” “I like to use new technologies,” and “It is a way to protect my community”—because of their theoretical grounding in TAM and their implications for shaping public perceptions and promoting broad adoption of future health technologies. Respondents were also asked why they might not want to use a portable electronic COVID-19 vaccine record like WA Verify, with 13 possible choices provided, and were again instructed to select all that applied. This analysis focused on 8 of these reasons: “Adding a tool like this to my phone would be too difficult,” “I don't carry my phone everywhere with me,” “My phone is too old for this kind of technology,” “I see no personal benefit; I don’t need to show my vaccination record to do the things I want to do,” “I’m concerned about data security when it comes to personal health data like this,” “I’m concerned that my phone would be more likely to be hacked,” “I don’t want public health authorities to have access to my personal data,” and “I worry that it would be easy to track me if this tool were on my phone.” These were again selected for their theoretical grounding in the TAM and to investigate exogenous, upstream factors of perceived usefulness and ease of use, particularly those related to misconceptions and negative perceptions that may affect health technology adoption.

Respondents were also asked the following question: “How would you like to receive information about WA Verify or similar tools that aim to support public health efforts to improve the health of all communities in our state?” The provided options were “During a COVID-19 vaccination appointment”, “Friends, family, or colleagues”, “Social media”, “News”, “Work/School”, “Health care provider”, “From a business or organization”, “While attending an event”, and “Some other way (please specify)”. Respondents could select “yes” for as many options as they wished. For this analysis, an additional variable, “None of these ways,” was created to capture those who did not select “yes” for any of the provided options for receiving WA Verify information.

“Tech readiness” was compared across user status groups. This measure, developed by the Pew Research Center to identify individuals who may be less prepared to use technology effectively [25,29], was slightly modified to fit the WA Verify survey questions. Respondents were classified as having “lower tech readiness” if they were either “not at all confident” or “only a little confident” using computers, smartphones, or other electronic devices to do things online, *or* if they said it was “true” or “very true” that they usually need help using new devices. “Higher tech readiness” was assigned to respondents who were “very confident” or “somewhat confident” using digital devices to do things online *and* who said it was “slightly true” or “not true at all” that they usually need help using new devices.

For survey items with write-in options, open-ended responses were reviewed and assigned to existing response categories where appropriate. This process ensured that all responses were comprehensively captured in the analysis.

### Sociodemographic, Health, and Technology Access and Engagement Characteristics

Survey respondents answered questions about sex/gender, age, race and ethnicity, education, disability status, language spoken at home, whether they received any vaccinations in the past 2 years, smartphone ownership, and awareness of WA Verify before completing the survey. Based on these responses, characteristics of the survey population were compared with WA State estimates from the United States Census Bureau 2021 5-year American Community Survey (ACS) [30,31].

### Creation and Implementation of WA State Poststratification Weights

This analysis used poststratification weights to reduce bias resulting from nonresponse in the survey sample and to obtain more representative findings [32]. Weights were created and applied so that the weighted population matched WA State marginally with respect to race/ethnicity and joint age-sex distributions. For respondents with missing data on age, sex, or combined race/ethnicity, a hot-deck imputation strategy was used, replacing missing values with observed responses from similar respondents [33]. Of note, imputed values were used solely for weight creation. Because respondent characteristics were unknown until after survey distribution, poststratification was applied to align the weighted marginal sample distributions with those of WA State [34]. As the survey used a household-level sample, weights accounted for household size, meaning each respondent represented their entire household. WA State margins were based on 2021 5-year ACS estimates [30]. Weights were created specifically for the present analytical population. Overall, 1389 out of 1401 (99.14%) respondents had weights below 5, and 1362 out of 1401 (97.22%) had weights below 3. Additional information and weight diagnostics are provided in Multimedia Appendix 2.

### Data Analysis

Counts and unweighted proportions were calculated for all sociodemographic characteristics and user status groups. Unweighted proportions for WA State from the 2021 5-year ACS were also calculated and presented side-by-side for comparison [30]. Counts, as well as unweighted and weighted proportions, were calculated for user status groups. For comparisons of support for COVID-19 protection policies, reasons for using or not using a tool like WA Verify, communication preferences regarding WA Verify information, and tech readiness, only weighted proportions were calculated and presented by user status group. The analysis focused on describing differences in attitudes and perceptions by user status; therefore, hypothesis testing, 95% CIs, and *P* values were not used to avoid implying causal relationships.

The analysis dataset was relatively complete, with very few records containing missing data. For feelings toward COVID-19 policies, less than 1% (12/1401, 0.86%) of the analytical population did not respond to this question. Questions on reasons for using or not using WA Verify and tech readiness appeared before those used to determine user status. Therefore, a complete case analysis was conducted. Missingness for demographic characteristics is presented in Table 1.

All analyses and data visualizations were performed using R version 4.3.2 (R Foundation), and the 2021 5-year ACS estimates were accessed via the tidycensus R package [35,36].

### Ethical Considerations

In June 2022, the UW Human Subjects Division reviewed the WA Verify evaluation project (Institutional Review Board ID STUDY00015786) and determined that it did not qualify as human subjects research under federal and state regulations. As such, the project was exempt from UW Institutional Review Board review. The Washington State University/Social and Economic Sciences Research Center Institutional Review Board subsequently confirmed this status.

Although the project was not classified as human subjects research, ethical and data privacy best practices were followed throughout. Data collection was designed to protect participant privacy and confidentiality, with minimal identifiers included. These identifiers were limited to broad demographic classifications. To encourage participation, cash incentives were provided as described above.

## Results

### Description of Survey Population

A total of 1491 individuals completed the WA Verify Statewide Survey, resulting in a response rate of 31.74% [(1440 completed + 51 partially completed)/(5000 invitations – 302 undeliverable surveys)] [37]. This analysis included 1401 respondents (n=90 excluded due to missing data on required user status questions). Of the 1401 respondents, 359 (25.6% unweighted, 25.8% weighted) were users, 662 (47.3% unweighted, 49.8% weighted) were potential users, and 380 (27.1% unweighted, 24.4% weighted) were unlikely users. All percentages reported are based on weighted data. After applying WA State poststratification weights, these percentages changed slightly to 25.8% users, 49.8% potential users, and 24.4% unlikely users (Figure 3). The STROBE (Strengthening the Reporting of Observational Studies in Epidemiology) checklist is presented as Multimedia Appendix 3.

Unweighted sociodemographic, health, and technology access and engagement characteristics of the analytic sample, stratified by user status group, are shown in Table 1, along with corresponding WA State statistics to assess representativeness, where available. User status groups showed similar distributions of self-reported race/ethnicity. Compared with users, unlikely users were more likely to be older (70+ years), report a disability, prefer not to specify their sex/gender, and live alone, but less likely to have at least a college degree, speak a non-English language at home, or own a smartphone. They were also more likely to report not having received any vaccines in the past 2 years. Potential users had characteristics that, on average, fell between those of users and unlikely users. However, regarding awareness of WA Verify, potential users aligned more closely with unlikely users, with 226 out of 662 (34.1%) potential users and 107 out of 380 (28.2%) unlikely users being aware of WA Verify before the survey. Compared with the WA State population, survey respondents were less likely to identify as male and were more likely to be over the age of 60 but less likely to be under 40 years of age. They were also more likely to have at least a college degree. Disability status was similar to that of WA State overall. White individuals were overrepresented, while Hispanic/Latinx individuals of any race were underrepresented in the survey sample compared with the state.

**Table 1:** Unweighted sociodemographic, health, and technology access and engagement characteristics of the analytical sample and comparison to WA State.

Variables		Users (n=359), n (%)	Potential users (n=662), n (%)	Unlikely users (n=380), n (%)	Overall (n=1401), n (%)	WA State (%)^a^
**Sex/Gender**						
	Female	212 (59.1)	378 (57.1)	193 (50.8)	783 (55.9)	50.0
	Male	144 (40.1)	258 (39.0)	141 (37.1)	543 (38.8)	50.0
	Transgender	0 (0)	3 (0.5)	0 (0)	3 (0.2)	N/A^b^
	Nonbinary/nonconforming	1 (0.3)	3 (0.5)	1 (0.3)	5 (0.4)	N/A
	Prefer not to respond	1 (0.3)	9 (1.4)	32 (8.4)	42 (3.0)	N/A
	Missing	1 (0.3)	11 (1.7)	13 (3.4)	25 (1.8)	N/A
**Age group (years)**						
	18-29	29 (8.1)	80 (12.1)	25 (6.6)	134 (9.6)	20.7
	30-39	67 (18.7)	104 (15.7)	44 (11.6)	215 (15.3)	19.0
	40-49	74 (20.6)	88 (13.3)	40 (10.5)	202 (14.4)	16.3
	50-59	65 (18.1)	104 (15.7)	52 (13.7)	221 (15.8)	16.0
	60-69	77 (21.4)	141 (21.3)	84 (22.1)	302 (21.6)	15.0
	70-79	36 (10.0)	103 (15.6)	84 (22.1)	223 (15.9)	8.7
	80+	11 (3.1)	32 (4.8)	35 (9.2)	78 (5.6)	4.3
	Missing	0 (0)	10 (1.5)	16 (4.2)	26 (1.9)	N/A
**Race/ethnicity**						
	American Indian/Alaska Native alone	0 (0)	3 (0.5)	1 (0.3)	4 (0.3)	0.9
	Asian alone	42 (11.7)	51 (7.7)	14 (3.7)	107 (7.6)	8.9
	Black alone	7 (1.9)	22 (3.3)	5 (1.3)	34 (2.4)	3.7
	Hispanic/Latinx any race	15 (4.2)	43 (6.5)	15 (3.9)	73 (5.2)	13.2
	Native Hawaiian and Other Pacific Islander alone	1 (0.3)	4 (0.6)	1 (0.3)	6 (0.4)	0.6
	Two or more races specified	16 (4.5)	20 (3.0)	22 (5.8)	58 (4.1)	5.8
	Some other race alone	1 (0.3)	5 (0.8)	10 (2.6)	16 (1.1)	0.4
	White alone	264 (73.5)	489 (73.9)	270 (71.1)	1023 (73.0)	66.5
	Missing	13 (3.6)	25 (3.8)	42 (11.1)	80 (5.7)	N/A
**Education**						
	Less than high school	1 (0.3)	5 (0.8)	6 (1.6)	12 (0.9)	8.1
	High school graduate	22 (6.1)	85 (12.8)	70 (18.4)	177 (12.6)	21.8
	2-year degree or some college	81 (22.6)	165 (24.9)	122 (32.1)	368 (26.3)	32.8
	4-year degree or more	255 (71.0)	393 (59.4)	164 (43.2)	812 (58.0)	37.3
	Missing	0 (0)	14 (2.1)	18 (4.7)	32 (2.3)	N/A
**Disability status**						
	Yes	36 (10.0)	80 (12.1)	68 (17.9)	184 (13.1)	13.7
	No	313 (87.2)	537 (81.1)	257 (67.6)	1107 (79.0)	86.3
	Prefer not to respond	10 (2.8)	32 (4.8)	40 (10.5)	82 (5.9)	N/A
	Missing	0 (0)	13 (2.0)	15 (3.9)	28 (2.0)	N/A
**Speak a language other than English at home**						
	Yes	58 (16.2)	76 (11.5)	31 (8.2)	165 (11.8)	20.3
	No	300 (83.6)	566 (85.5)	327 (86.1)	1193 (85.2)	79.7
	Missing	1 (0.3)	20 (3.0)	22 (5.8)	43 (3.1)	N/A
**Vaccinated in the past 2 years**						
	Yes	358 (99.7)	633 (95.6)	295 (77.6)	1286 (91.8)	N/A
	No	0 (0)	16 (2.4)	63 (16.6)	79 (5.6)	N/A
	Missing	1 (0.3)	13 (2.0)	22 (5.8)	36 (2.6)	N/A
**Smartphone ownership**						
	Yes	357 (99.4)	647 (97.7)	325 (85.5)	1329 (94.9)	N/A
	No	2 (0.6)	12 (1.8)	51 (13.4)	65 (4.6)	N/A
	Missing	0 (0)	3 (0.5)	4 (1.1)	7 (0.5)	N/A
**Aware of WA Verify before survey**						
	Yes	307 (85.5)	226 (34.1)	107 (28.2)	640 (45.7)	N/A
	No	52 (14.5)	434 (65.6)	270 (71.1)	756 (54.0)	N/A
	Missing	0 (0)	2 (0.3)	3 (0.8)	5 (0.4)	N/A
**Mode of survey completion**						
	Mail	65 (18.1)	131 (19.8)	117 (30.8)	313 (22.3)	N/A
	Web	294 (81.9)	531 (80.2)	263 (69.2)	1088 (77.7)	N/A

^a^Per United States Census Bureau American Community Survey (ACS) 2021 5-year estimate (Census Bureau, 2022) [31]. ACS tables used for statistics were B01001 (sex/gender and age), B03002 (race/ethnicity), B15003 (education), B18101 (disability status), and B16001 (speak language other than English at home).

^b^N/A: not applicable.

### Support for Policies That Require Proof of Vaccination or Test Result

**Figure 4:**
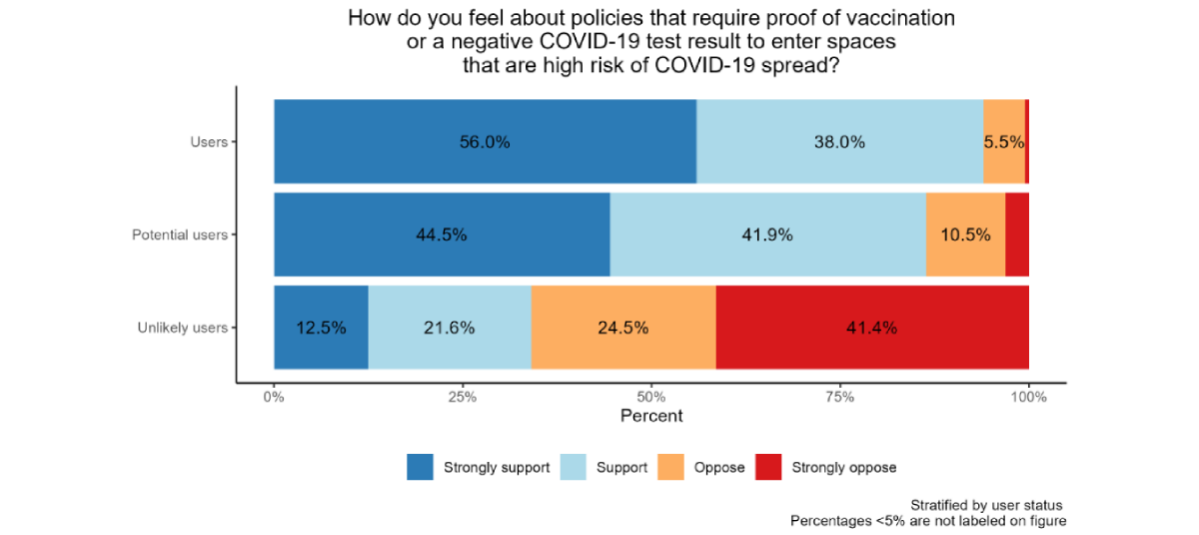
Distribution of opinions regarding proof of COVID-19 vaccination and negative test policies by user status. All percentages have poststratification weights incorporated as described.

All results reported below incorporate WA State poststratification weights. Users were overwhelmingly supportive, with 56.0% strongly supporting and 38.0% supporting policies that require proof of vaccination or a test result. Only 5.5% of users reported opposing these policies, with less than 1% expressing strong opposition. Potential users followed closely, with 44.5% reporting strong support and 41.9% reporting support. Only 3.1% of potential users reported strong opposition. Unlikely users mostly opposed the policies, with 41.4% strongly opposing and 24.5% opposing. Only 12.5% strongly supported, and 21.6% supported the policies. Figure 4 presents the weighted distribution of opinions regarding proof of COVID-19 vaccination and negative test policies by user status.

### Reasons to Use WA Verify

**Figure 5:**
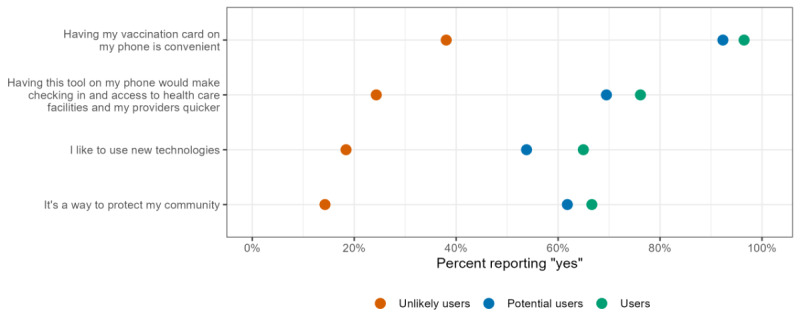
Distribution of reasons to use a portable electronic COVID-19 vaccine record like WA Verify by user status. All percentages have poststratification weights incorporated as described.

Reasons cited for wanting to use a tool like WA Verify varied by user status group. For the reason “Having my vaccination card on my phone is convenient,” a vast majority of both users and potential users agreed—96.5% and 92.3%, respectively. By contrast, only 38.1% of unlikely users selected “yes.” Regarding the reason “Having this tool on my phone would make checking in and access to health care facilities and my providers quicker,” 76.2% of users, 69.5% of potential users, and 24.3% of unlikely users agreed. For “I like to use new technologies,” 65.0% of users and 53.8% of potential users responded “yes,” while only 18.4% of unlikely users did. Regarding the reason “It is a way to protect my community,” 66.6% of users and 61.8% of potential users agreed. Only 14.3% of unlikely users cited this as a reason to use a tool like WA Verify. Figure 5 shows weighted distributions by user status. Only the weighted percentages of those reporting “yes” are presented in the figure, as the response options were limited to “yes” or “no.”

### Reasons to Not Use WA Verify

Unlikely users were more likely to cite the presented options as reasons for choosing not to use a tool like WA Verify. For the reason “I’m concerned about data security when it comes to personal health data like this,” 20.7% of users, 33.9% of potential users, and 65.7% of unlikely users responded “yes.” “I don’t want public health authorities to have access to my personal data” showed the largest gap between unlikely users and both users and potential users, in both absolute and relative terms: 10.3% of users and 20.6% of potential users cited this as a reason not to use WA Verify, compared with 63.4% of unlikely users. For “I worry that it would be easy to track me if this tool was on my phone,” 12.6% of users, 26.6% of potential users, and 53.9% of unlikely users responded “yes.” Results for “I’m concerned that my phone would be more likely to be hacked” followed a similar pattern: 8.8% of users, 13.8% of potential users, and 40.4% of unlikely users responded “yes” as a reason not to use WA Verify. Overall, reasons related to technology were less prevalent than those related to security and data privacy concerns. However, differences between groups emerged: 36.9% of unlikely users reported not carrying their phones everywhere, compared with only 10.4% of potential users and 7.3% of users. For the reason “Adding a tool like this to my phone would be too difficult,” 5.5% of users, 8.6% of potential users, and 21.2% of unlikely users responded “yes.” Few respondents reported that their phone was too old for technology such as WA Verify, though this concern was highest among unlikely users at 13.2%. Finally, a majority (63.1%) of unlikely users reported seeing “no personal benefit” in using a tool like WA Verify, as they do not need to show their vaccination record to do the things they want to do. Prevalences were 22.3% among potential users and 9.0% among users.

**Figure 6:**
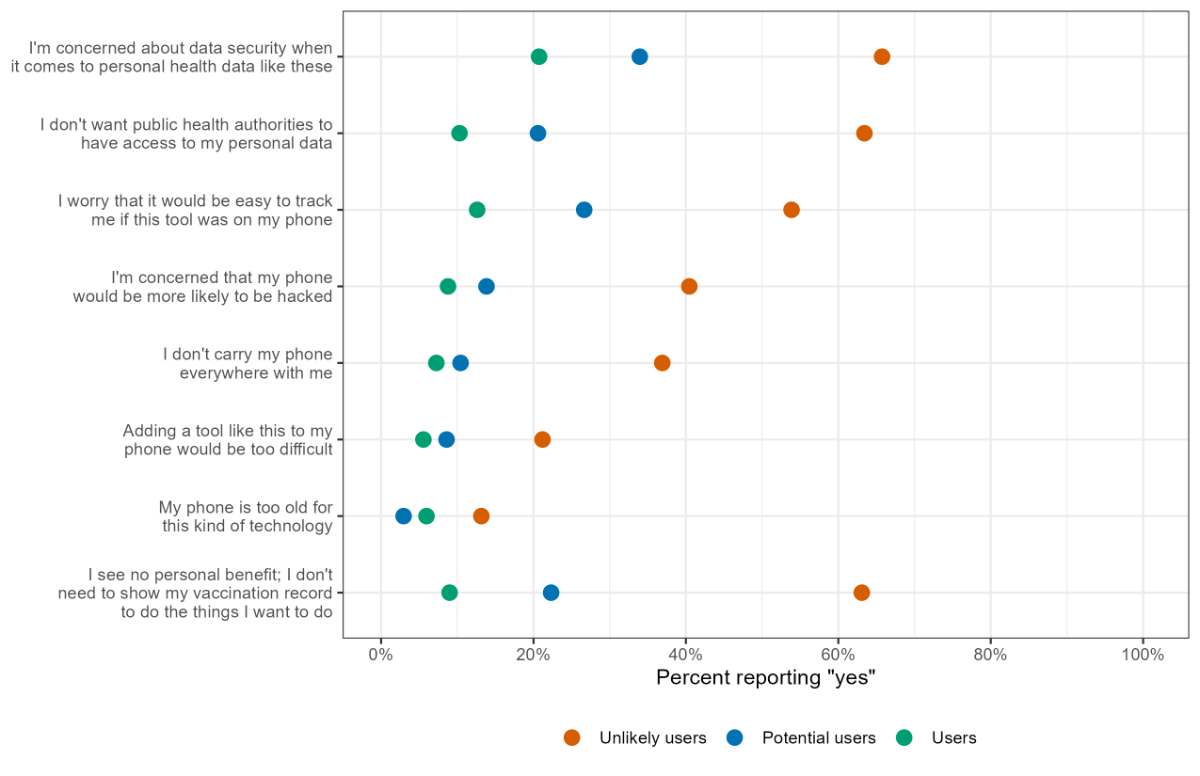
Distribution of reasons to not use a portable electronic COVID-19 vaccine record like WA Verify by user status. All percentages have poststratification weights incorporated as described.

Figure 6 shows the weighted distribution of reasons for not using WA Verify by user status, presenting only the percentages of respondents who answered “yes” to each reason.

### WA Verify Information Communication Preferences

**Figure 7:**
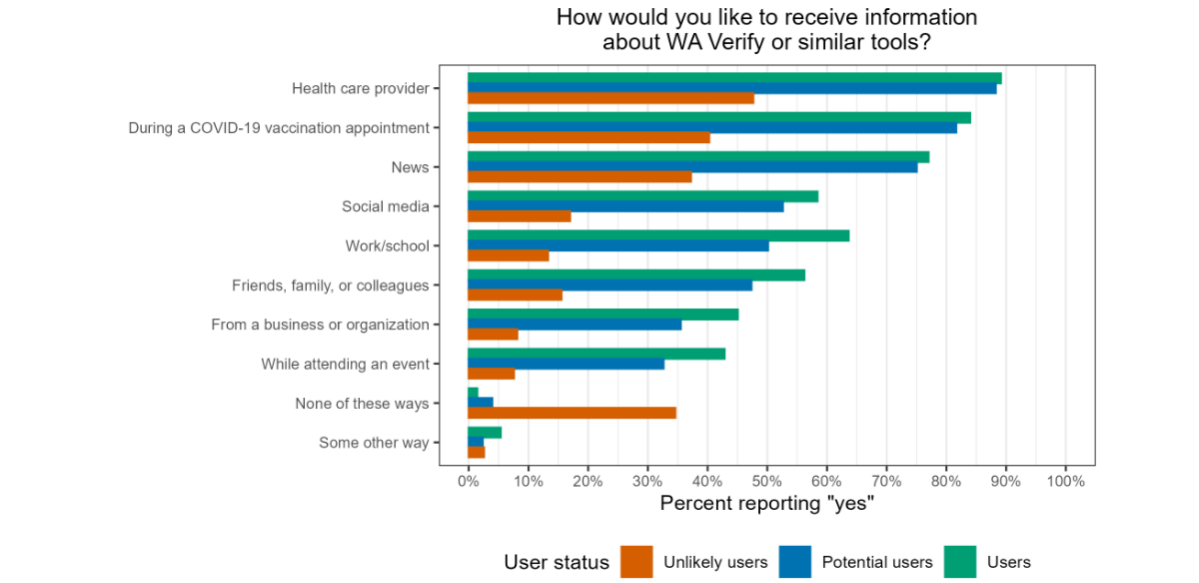
Distribution of preferred information sources for WA Verify or similar tools stratified by user status. All percentages have poststratification weights incorporated as described.

Regarding how respondents would like to receive information about WA Verify or similar tools, the most common source was a health care provider, followed by during a COVID-19 vaccine appointment, and then from the news across all 3 user groups. However, there were stark differences for unlikely users. For example, 89.1% of users and 88.3% of potential users selected “health care provider” as a preferred information source, compared with only 47.7% of unlikely users. No single option was selected by a majority of unlikely users, and 34.6% of this group did not select any of the provided communication options, including “some other way.” By contrast, only 3.9% of potential users and 1.4% of users did not select any communication options. Figure 7 shows the weighted distribution of preferred information sources through which respondents would like to receive information about WA Verify or similar tools.

### Tech Readiness

Overall, 19.0% of the total population (regardless of user status) was classified as having “lower tech readiness.” This percentage was lowest among users at 12.3%, slightly higher among potential users at 15.9%, and highest among unlikely users at 32.3% (data not shown).

## Discussion

### Principal Findings and Findings in Context

This analysis explored perceptions, characteristics, and barriers to adopting a VCI tool, investigating if and how these factors relate to willingness to use a digital public health tool like WA Verify, an established COVID-19 vaccine verification system in WA State. The results provide insights into the main barriers to adoption among those most likely to be resistant or hesitant adopters. These findings are essential for developing effective communication strategies to improve uptake and ensure equity in access to digital public health technologies. While evaluation of COVID-19 vaccine verification technology has largely concluded, the findings and their implications remain applicable to other efforts aimed at understanding barriers, facilitators, and perceptions related to future public health informatics tools.

Potential users of WA Verify or similar tools likely constitute the plurality of WA State residents. Overall, this group’s sociodemographic characteristics, policy attitudes, reasons for using or not using a VCI tool, public health communication preferences, and technological engagement and readiness were similar to those of self-reported WA Verify users. This is encouraging, as it suggests that many who did not use WA Verify did not opt out due to ideological reasons or strong opposition to the technology. Rather, they may have been less aware of this QR code–based vaccine verification system or faced logistical or technical barriers—both of which are supported by the results.

However, even among WA Verify users and potential users, concerns about data security, privacy of personal health information, phone hacking, and tracking were significant. Although these concerns may not be enough to prevent these groups from using VCI tools, they highlight the need for enhanced transparency and improved public health communication around data protection measures. Perhaps unsurprisingly, unlikely users were far more likely to cite concerns about data security, privacy, hacking, and tracking as reasons for not using WA Verify, indicating that these populations would require more outreach and assurances to build trust and acceptance. Previous research has shown that trust, privacy, and security concerns are common barriers to willingness to share health data with third parties [24], and such concerns around COVID-19 personal health information likely influence willingness to use contact tracing apps [38]. If an individual believes their health data are not secure, this perception is likely to reduce their view of the technology’s usefulness, ultimately lowering adoption potential, as posited by the TAM [15,23]. Concerns about data privacy, security, and general distrust in institutions responsible for storing such data—particularly regarding “vaccine passports” and digital COVID-19 contact tracing applications—emerged during the pandemic [39-41]. In a qualitative study from the United Kingdom, authors reported that participants’ acceptance of COVID-19 vaccine passports depended on the government’s ability to securely handle and use their health data, which was influenced by participants’ feelings toward political leaders [42]. Similarly, a mixed methods study in Montréal, Canada, found that while vaccine passports likely increased COVID-19 vaccine uptake, they may have also fueled distrust of scientific and governmental institutions among adolescents [43]. A study conducted in June 2020—before widespread discussions about or the rollout of COVID-19 vaccines—that surveyed a nationally representative sample of Americans reported a roughly even split between support and opposition to COVID-19 “immunity privileges.” Surprisingly, overall support did not vary by political affiliation, though there was greater support for privately administered certificates compared with government-issued “passports” [44]. While empirical evidence on perceptions of vaccine passport technology in the United States remains limited—likely due to policy variations across jurisdictions often aligned with partisan affiliation—the COVID-19 vaccine rollout prompted significant discussions around the legal, ethical, security, and privacy aspects of COVID-19 and other health data [39,45-49]. Addressing these concerns and fostering trust will be critical to ensuring the successful adoption of digital public health tools.

Health care providers and COVID-19 vaccination appointments were the 2 most frequently chosen sources from which respondents across all user groups wanted to receive information about WA Verify or similar VCI tools. This aligns with other research on the American public’s communication preferences and trust in health information. A survey conducted in February 2022 found that health care professionals—specifically doctors and nurses—were the 2 most trusted sources for health improvement information among 17 options [50]. In the present analysis, users and potential users expressed interest in receiving information about WA Verify or similar tools through multiple channels. As many as 5 of the 8 specified survey options were selected by a majority in these groups, suggesting that future promotional campaigns could effectively drive adoption regardless of which information sources are utilized. By contrast, unlikely users showed far less interest in receiving information through any channel, with no single source selected by a majority and over one-third of respondents choosing none of the provided options. These varying preferences across user groups highlight the importance of targeted social marketing campaigns that segment audiences based on their beliefs, perceptions, and willingness to engage with health technologies such as VCI tools [51-53]. Social marketing campaign research during the COVID-19 pandemic often focused on promoting vaccine uptake [54,55], encouraging use of at-home antigen tests [56], and fostering protective behaviors such as mask-wearing and social distancing, including efforts in WA State in partnership with a local communications agency [57,58]. Findings from this analysis may inform content development for social marketing campaigns aimed at increasing future VCI tool uptake, laying the groundwork for audience segmentation strategies based on user group categorizations that could more effectively drive adoption.

A vast majority of users and potential users reported that having vaccination cards on their phones was convenient, and a slightly smaller—but still substantial—majority in these groups said that having WA Verify on their phone would speed up access to health care facilities and providers. Additionally, few users and potential users reported seeing no personal benefit in using WA Verify. Responses from unlikely users diverged, reflecting aspects of the TAM framework [15]. The agreement among users and potential users that WA Verify provides convenient access to vaccination cards and personal benefits indicates high perceived ease of use and usefulness—likely influenced by their relatively high comfort with new technologies. This was not the case among unlikely users; fewer than one-fifth of this group reported liking to use new technologies as a reason to adopt a tool like WA Verify. The analysis also showed that unlikely users were more likely to state that adding a tool like WA Verify to their phone would be too difficult, had lower tech readiness [25], and were far more likely to report not owning a smartphone and to choose the mail-based survey option compared with users and potential users. These external “technology comfort” factors, or antecedents in the TAM, likely contribute to the observed differences in perceived usefulness and ease of use of WA Verify among the populations. Poor computer skills and low technology self-efficacy have been linked to lower acceptance of general and health technologies, particularly among older adults, which may help explain the outcomes seen here [12,59,60]. Individuals with lower tech readiness and proficiency may be less likely to adopt new public health technologies, potentially limiting their access to the associated benefits. Notably, 13% of unlikely users reported not owning a smartphone, which could partly explain their disinterest due to a lack of access to the necessary device. These findings highlight the importance of addressing the digital divide and the risk that public health initiatives could inadvertently exacerbate existing inequities. It is crucial to proactively consider how new public health technologies might exacerbate existing digital health disparities, ultimately affecting downstream health and health care outcomes [61-63]. The digital health divide is shaped by individual and intersecting social determinants—such as poverty, lower health and digital literacy, lack of motivation to use new technology, and limited access—that also contribute to broader health disparities [64]. Providing educational materials, targeted social marketing outreach, and tutorials on using emerging public health technologies—tailored to audiences with lower tech readiness—may help bridge the digital divide. Additionally, ensuring nondigital alternatives are always available for those without smartphones or necessary technology is essential for equity. Finally, although improving access to smartphones, internet, and technology may not fall directly under public health practitioners’ responsibilities, advocating for these issues and highlighting their links to health disparities can drive meaningful progress.

“Vaccine passports” and vaccine verification processes are not new in public health. Under International Health Regulations, countries may require international travelers to show proof of vaccination for certain diseases—such as yellow fever—using an International Certificate of Vaccination or Prophylaxis [65]. Additionally, most US states require proof of meningitis vaccination for college or university enrollment due to the increased outbreak risk in these settings [66,67]. However, the COVID-19 pandemic changed how vaccine verification is used in the United States; proof of COVID-19 vaccination or negative tests became necessary for domestic travel, access to community spaces, and participation in everyday activities—though requirements varied inconsistently across cities and states. The daily and widespread use of COVID-19 vaccine verification likely fueled additional controversy, sparking socioethical debates alongside circulating conspiracy theories about the vaccine itself [42,68,69]. It is noteworthy that nearly one-fourth of unlikely WA Verify users reported no vaccinations in the past 2 years, compared with under 5% in the other 2 groups. Given that the final surveys were returned in January 2023, it is reasonable to assume that this nearly one-fourth had not received a COVID-19 vaccine. Having never been vaccinated—or feeling ethically opposed to the COVID-19 vaccine or vaccines more broadly—could complicate efforts to promote widespread use of VCI tools, reflecting ongoing public health challenges that have emerged and intensified since the pandemic began [70-72].

### Strengths and Limitations

This analysis had several strengths. First, data were drawn from a survey distributed to a random sample of all residential households within WA State, rather than a convenience sample, likely enhancing representativeness and diversity of respondents. However, the results may not be generalizable to populations outside WA State, as underlying demographics and experiences with vaccine verification technologies can vary between states. Both web-based and mailed survey options were offered, likely broadening the range of respondents—particularly in terms of tech readiness. Additionally, minimal missing data suggest that respondents did not find the survey overly burdensome, allowing for stronger inferences.

This analysis also had several limitations. First, all survey data were self-reported, which may have introduced information, recall, and social desirability biases. Although the survey explained what WA Verify is and how it is used, respondents might have forgotten prior use of WA Verify or confused it with other health technology tools that were emerging during this period. Respondents may also have overstated their confidence in using or setting up electronic devices. Additionally, the survey’s overall response rate was 1491 out of 4698 (31.74%), which is suboptimal and could introduce bias. However, applying WA State poststratification weights likely improved representativeness, enhancing the validity and generalizability of the findings. Furthermore, this was a cross-sectional survey, limiting causal inferences due to temporal ambiguity. For example, it is not possible to determine whether an unlikely user’s attitude toward vaccine verification and negative test policies existed before the COVID-19 pandemic or if strong opposition developed in response to encountering a tool like WA Verify. Finally, while the survey population generally reflected the demographic characteristics of WA State, the sample size was insufficient to precisely characterize perceptions among non-White and non-Asian individuals. Given the historical and ongoing concerns about data tracking in Black and Brown communities—fueled by government surveillance and health disparities [73-75]—it is crucial to collaborate directly with these communities to refine messaging, build trust, and effectively address their concerns.

### Future Directions

While COVID-19 vaccination verification has become less common, understanding public perceptions of VCI, QR code–based, and other digital health verification tools will support the rollout of future initiatives for sharing health information. In fact, multiple representatives of the Pan American Health Organization recently issued an urgent call advocating for widespread use of digital vaccine certificates to “improve health outcomes, ensure equitable access to health services, and enhance the overall efficiency of health systems in the Americas,” underscoring the importance and timeliness of this work and its future expansions [76]. For example, WA DOH is currently developing a platform to expand on WA Verify, enabling individuals to manage and share personal health information—including immunizations, medications, and clinical histories—via QR codes or shareable web links with health care providers, family, and others [77]. This system will utilize International Patient Summary standards and SMART Health Links to ensure feasibility, verifiability, and optimal data security [6,78]. Another WA DOH initiative is exploring the use of this technology for Portable Orders for Life-Sustaining Treatment forms—which document end-of-life wishes for individuals in poor health during medical emergencies—and other advance directives [79,80]. Other future applications that could benefit the public include using SMART Health Links to provide access to advance directives, complete immunization records, laboratory results for notifiable conditions, and medical records while traveling. To ensure equitable access and reach diverse population segments, all of these options should be offered in multiple languages; notably, WA Verify was available in 52 languages. However, the process of translating WA Verify was time-intensive and could be streamlined with the use of artificial intelligence. Nonetheless, several security, ethical, and equity considerations must be addressed before this approach can be widely adopted [81]. Verification tools may be especially valuable for managing childhood vaccination records in WA State. A 2019 state policy change that eliminated personal belief exemptions for the MMR (measles-mumps-rubella) vaccine resulted in increased completion rates of the MMR series and a decrease in overall exemption rates. This precedent highlights how verification tools could be expanded to support and encourage parental compliance with vaccination requirements [82,83]. Understanding why WA State residents—particularly potential and unlikely users—are hesitant to use VCI tools can guide strategies for system design, rollout, and targeted communication. This insight is crucial for improving adoption rates and building public trust, especially around concerns such as data security. Additionally, the absence of follow-up with individual survey participants limited our ability to explore what might encourage potential and unlikely users to adopt tools like WA Verify. Further research into factors that could motivate these groups to embrace VCI tools would be valuable and could help guide more effective public health strategies.

### Conclusions

This analysis examined perceptions, attitudes, barriers, and facilitators related to the uptake of a VCI tool—specifically WA Verify—in WA State, and investigated how these variables differ based on willingness to use such a tool. Nearly three-quarters of WA State residents were either interested in using or already using this VCI tool, making users and potential users ideal targets for adoption promotion. However, concerns about data security and distrust of public health and its policies were common and posed significant obstacles to widespread acceptance and use, particularly among unlikely users. Additionally, lower technological proficiency presents a barrier to acceptance and uptake, underscoring the need for targeted outreach and education to ensure equitable access to public health technologies. Understanding misperceptions and barriers among those most likely to be hesitant is crucial for public health efforts to develop tailored and effective communication strategies. These findings provide valuable insights to guide the design and implementation of future public health tools, ultimately improving user engagement.

## Appendix

Multimedia Appendix 1 Survey instrument.

Multimedia Appendix 2 Additional diagnostics for the Washington State population weights created and implemented in the analysis.

Multimedia Appendix 3 STROBE checklist.

## Abbreviations

ACS: American Community Survey
MMR: measles-mumps-rubella
STROBE: Strengthening the Reporting of Observational Studies in Epidemiology
TAM: Technology Acceptance Model
VCI: Verifiable Clinical Information
WA: Washington
WA DOH: Washington Department of Health

## Data Availability

In accordance with WA DOH policies, the statewide survey data analyzed in this study are not publicly available at this time.
