# Supplementary material for: Exploring Attitudes and Obstacles Around Digital Public Health Tools: Insights From a Statewide Cross-Sectional Survey on Washington’s Vaccine Verification System
Source: J Med Internet Res. 2025 Oct 3;27:e66550. doi: 10.2196/66550 (PMC12534757; doi:10.2196/66550)
Supplement: Multimedia Appendix 1 [file jmir_v27i1e66550_app1.pdf]

SCHOOL OF PUBLIC HEALTH

Public Health Technology in Washington

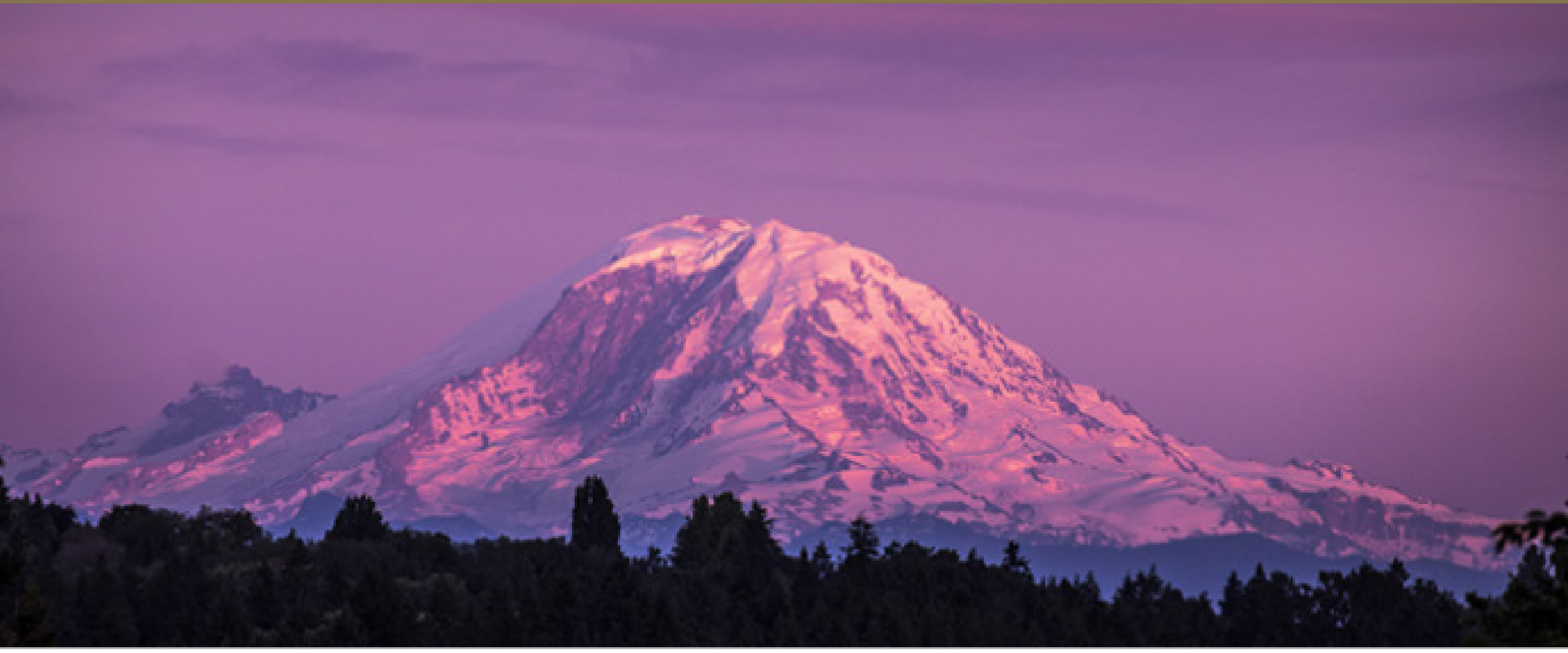

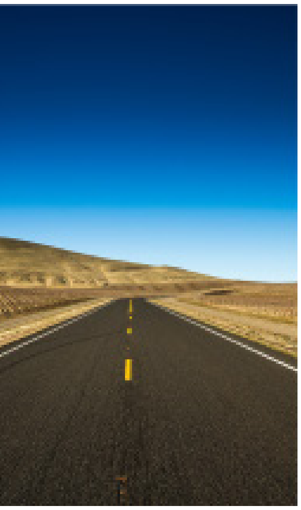

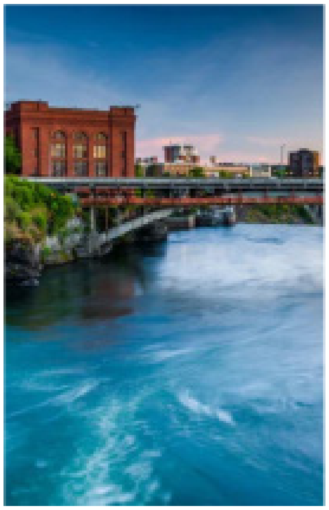

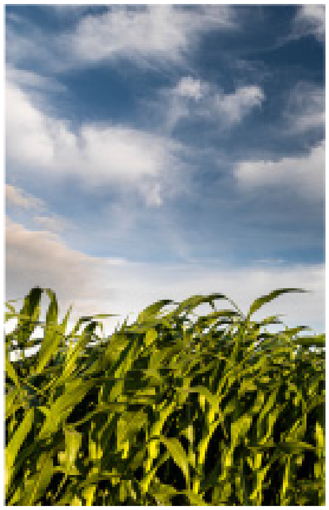

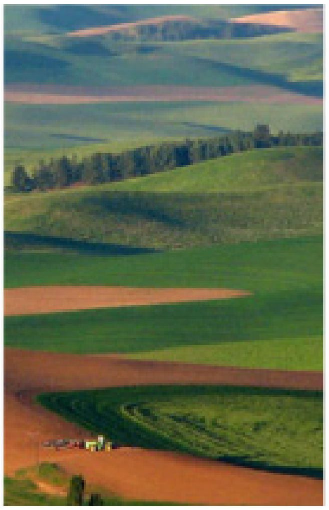

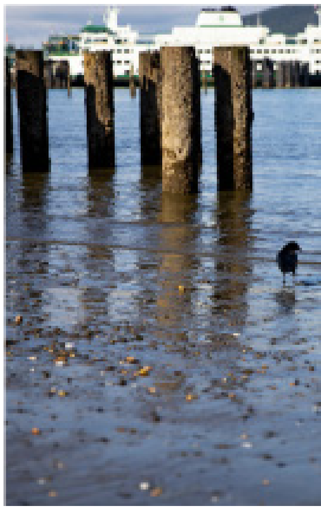

The University of Washington School of Public Health, in collaboration with Washington State University's Social and Economic Sciences Research Center, is conducting a survey to hear how Washington residents used technology during the pandemic. Results from this study will be used to help shape future public health efforts and develop useful tools to keep our Washington communities healthy.

Your household has been randomly selected to participate in this survey and we want to be sure the survey results truly represent adults living in our state. We ask that the adult, 18 years of age or older, who is currently living in your household and has had the most recent birthday, complete it.

☐ I agree. I am at least 18 years old and agree to participate in this survey.

The first section of this survey asks about your experiences with different technologies.

Q1. Overall, how confident, if at all, do you feel using computers, smartphones, or other electronic devices to do the things you need to do online?

① Very confident
② Somewhat confident
③ Only a little confident
④ Not at all confident

Q2. How true is the following statement? "When I get a new electronic device, or need to do a new task on it, I usually need someone else to set it up, show me how to use it, or help me."

① Very true
② True
③ Slightly true
④ Not at all true

Q3. Do you own a smartphone? *A smartphone is a mobile device (for example an iPhone or Android) that is capable of accessing the Internet and downloading apps.*

① Yes
② No

Q4. Do you or any member of your household have access to the Internet using a . . .
(Please select "Yes" or "No" for each row)

	Yes	No
Cellular data plan for a smartphone or other mobile device?	①	②
Broadband (high speed) Internet service such as cable, fiber optic or DSL service installed in this household?	①	②
Satellite internet service installed in this household?	①	②
Dial-up Internet service installed in this household?	①	②
Some other service, *(please list)*: ______________________	①	②

Q5. Have you ever used any electronic health tools? *(Please select "Yes" or "No" for each row)*

	Yes	No
An online patient medical record or health portal (for example MyChart)	①	②
WA Notify (WA's COVID-19 Exposure Notification Tool)	①	②
V-safe (CDC's post-vaccine health check tool)	①	②
A tool for keeping track of my activities like diet and exercise (for example Fitbit, Strava, or MyFitnessPal)	①	②
Other tools, *(please list)*: ______________________	①	②

Some businesses and groups have asked that a COVID-19 vaccination card or a negative COVID-19 test result is presented before allowing people to enter their building or use their services. In this section, we would like to learn about your experiences with showing proof of COVID-19 vaccination or COVID-19 test results.

Q6. In the last 12 months, have you been asked to show proof of COVID-19 vaccination or a negative COVID-19 test result before participating in an activity or entering a business?

① Yes
② No ⟶ **Skip to Q8**

Q7. In which of the following situations have you been asked to show proof of COVID-19 vaccination and/or COVID-19 test results? *(Please select "Yes" or "No" for each row)*

	**A. Proof of Vaccination**		**B. Test Results**	
	**Yes**	**No**	**Yes**	**No**
Going to my workplace	①	②	①	②
Going to school	①	②	①	②
Entering a health care facility	①	②	①	②
Attending an event, such as a concert or sporting event	①	②	①	②
Attending a social gathering	①	②	①	②
Attending a religious service	①	②	①	②
Entering a restaurant, bar, or club	①	②	①	②
Using a gym or fitness center	①	②	①	②
Booking or boarding a trip by plane, train, ferry or cruise ship	①	②	①	②
During interstate travel	①	②	①	②
During international travel	①	②	①	②
Another situation, *(please specify)*: ______________________	①	②	①	②

This next section focuses on WA Verify. WA Verify is a portable electronic COVID-19 vaccine record (provided by the Washington State Department of Health) that has been developed to store this information as a QR code. The QR code can be viewed on a smartphone or printed onto paper to verify vaccination status. A QR code is a machine-readable code made up of black and white squares like this . . .

We would like to hear your thoughts about this tool.

Q8. Before receiving this survey, had you heard about WA Verify?

① Yes
② No ⟶ **Skip to Q10**

Q9. How did you first hear about WA Verify?

1. Friends, family, or colleagues
2. Social media
3. News
4. Work/School
5. At a COVID-19 vaccination or testing appointment
6. Healthcare provider
7. Before attending an event or traveling
8. From a business or organization
9. While attending an event
10. Department of Health Hotline
11. Some other way, *(please specify)*: ____

Q10. We're interested in knowing why someone might choose to use a portable electronic COVID-19 vaccine record like WA Verify. Please select all the reasons you would use a tool like this.

	Yes	No
I'd rather not carry a paper COVID-19 vaccine card.	1	2
Having my vaccination card on my phone is convenient.	1	2
I always have my phone with me.	1	2
I travel so this tool would make check-in and other travel-related activities easy.	1	2
I need to show my vaccination record for work.	1	2
A paper vaccine card is too easy to lose.	1	2
I need to show my vaccination record for school.	1	2
It is a way to protect my community	1	2
A vaccination record on my phone makes it easier to get into social gatherings, activities, or events I want to attend.	1	2
I like to use new technologies.	1	2
Having this tool on my phone would make checking in and access to healthcare facilities and my providers quicker.	1	2
Another reason, *(please specify)*: ____	1	2

Q11. We're i nterested in learning more about why someone might not want to use a portable electronic COVID-19 vaccine record like WA Verify. Please select all the reasons you would not want to use a tool like this.

	Yes	No
I would prefer carrying a paper COVID-19 vaccine card.	①	②
Adding a tool like this to my phone would be too difficult.	①	②
I don't carry my phone everywhere with me.	①	②
I have no need to show my vaccination record for work or school.	①	②
My phone is too old for this kind of technology.	①	②
I have no need to show my vaccination record for travel.	①	②
I haven't been vaccinated so this tool is not useful.	①	②
I see no personal benefit; I don't need to show my vaccination record to do the things I want to do.	①	②
I'm concerned that my phone would be more likely to be hacked.	①	②
I don't want public health authorities to have access to my personal data.	①	②
I'm concerned about data security when it comes to personal health data like this.	①	②
I worry that it would be easy to track me if this tool was on my phone.	①	②
Another reason, *(please specify)*: ________________________________	①	②

Q12. Do you use WA Verify?

① Yes → **Skip to Q14**
② No
③ No, but I use another, similar tool, *(please specify)*: ____________________ → **Skip to Q14**

Q13. How willing would you be to use a portable electronic COVID-19 vaccine record?

① Willing
② Somewhat willing
③ Somewhat not willing
④ Not willing

In this section we would like to hear more about your views on how new technologies like WA Verify might be used.

Q14. How do you feel about policies that require proof of vaccination or a negative COVID-19 test result to enter spaces that are high risk of COVID-19 spread?

① I strongly support these policies
② I support these policies
③ I oppose these policies
④ I strongly oppose these policies

Q15a. To what extent do you agree or disagree with the following statements? Using new technologies like WA Verify…

	Strongly agree	Agree	Disagree	Strongly disagree
Will help limit the spread of COVID-19.	1	2	3	4
Could stigmatize people who are not vaccinated.	1	2	3	4
Will help improve the economy.	1	2	3	4
Could cause harm to people from marginalized communities.	1	2	3	4
Will help our lives return to "normal".	1	2	3	4
Only benefits people who have smartphones.	1	2	3	4
Helps protect individuals who can't be vaccinated for health reasons.	1	2	3	4
Could increase social and economic inequities.	1	2	3	4
Will benefit everyone, even those who have not been vaccinated.	1	2	3	4
Helps protect individuals who are at higher risk for severe COVID-19.	1	2	3	4
Excludes people who have lower digital skills.	1	2	3	4
Would make vaccine records more accessible.	1	2	3	4

Q15b. Please use this space to elaborate or expand on any of your answers above.

Q16. The Washington State Department of Health is trying to inform residents about WA Verify. How would you like to receive information about WA Verify or similar tools that aim to support public health efforts to improve the health of all communities in our state?

	Yes	No
During a COVID-19 vaccination appointment	1	2
Friends, family, or colleagues	1	2
Social media	1	2
News	1	2
Work/School	1	2
Healthcare provider	1	2
From a business or organization	1	2
While attending an event	1	2
Some other way, *(please specify)*: ________________________	1	2

We want to be sure sure we are reaching a diverse, representative sample of Washington residents with this survey. We appreciate you telling us a little more about yourself.

Q17. How old are you?

① 18-29
② 30-39
③ 40-49
④ 50-59
⑤ 60-69
⑥ 70-79
⑦ 80+

Q18. Are you . . . ?

① Female
② Male
③ Transgender
④ Non-binary/non-conforming
⑤ Prefer not to respond

Q19. How would you describe your race and ethnicity?

	Yes	No
American Indian-Alaskan Native (AIAN)	①	②
Asian	①	②
Black	①	②
Hispanic/Latinx	①	②
Native Hawaiian and Other Pacific Islander (NHOPI)	①	②
White	①	②
Another race or ethnicity. *(Please specify)*: ______________________________	①	②

Q20. Do you consider yourself to have a disability?

① Yes
② No, I don’t consider myself to have a disability
③ Prefer not to respond

Q21. Do you speak a language other than English at home?

① Yes ➔ Which language(s)? *(Please specify)*:______________________________
② No

Q22. Including yourself, how many people currently live in your household?

① One, I live alone
② Two
③ Three
④ Four
⑤ Five
⑥ Six or more

Q23. Are you a parent or guardian to a child under 18 years old?

① Yes
② No

Q24. What is your highest level of education?

① Less than high school
② GED (General Educational Development)
③ High school graduate
④ 2 year degree or some college
⑤ 4 year degree or more

Q25. Have you received any vaccinations in the past two years? *(For example: Influenza, Shingles, Pneumococcal, COVID-19.)*

① Yes
② No

Thank you for completing our survey.

Would you like to get paid to help with work we will be doing in the future? We would like to hear more about your views on WA Verify and other digital tools that can be used to limit the spread of COVID-19 and protect the health of Washington residents. If you choose to participate in future activities, you will be offered a gift card for your time.

To sign up, please visit: https://redcap.link/WAHealth
Or Scan the QR Code
