# Supplementary material for: Exploring Attitudes and Obstacles Around Digital Public Health Tools: Insights From a Statewide Cross-Sectional Survey on Washington’s Vaccine Verification System
Source: J Med Internet Res. 2025 Oct 3;27:e66550. doi: 10.2196/66550 (PMC12534757; doi:10.2196/66550)
Supplement: Multimedia Appendix 2 [file jmir_v27i1e66550_app2.docx]

**Supplemental Materials**

As described in the main body of the manuscript, non-response in our survey may have led to a non-representative sample of the state’s population. As a result, we created and implemented population weights so that the weighted population matched that of WA State, marginally, with respect to combined race/ethnicity and joint age-sex distributions. Additionally, respondent household size was also incorporated, meaning the respondent represented those in their household, as our survey was distributed at the household level.

Supplemental Figure 1 presents a histogram of the survey respondent weights in our analysis. Overall, 99.1% of respondents’ weights were below five, and 97.2% of respondents’ weights were below three. Supplemental Table 1 shows the weights, race/ethnicity, age, sex, and household size of the survey respondents with ten highest weights in our analysis. All respondents lived in households of four or more individuals and were between the ages of 18 and 29 years. Additionally, 80% identified as Hispanic of any race, and one individual was non-binary/gender non-conforming.

**Supplemental Figure 1**. Distribution of analytical survey weights


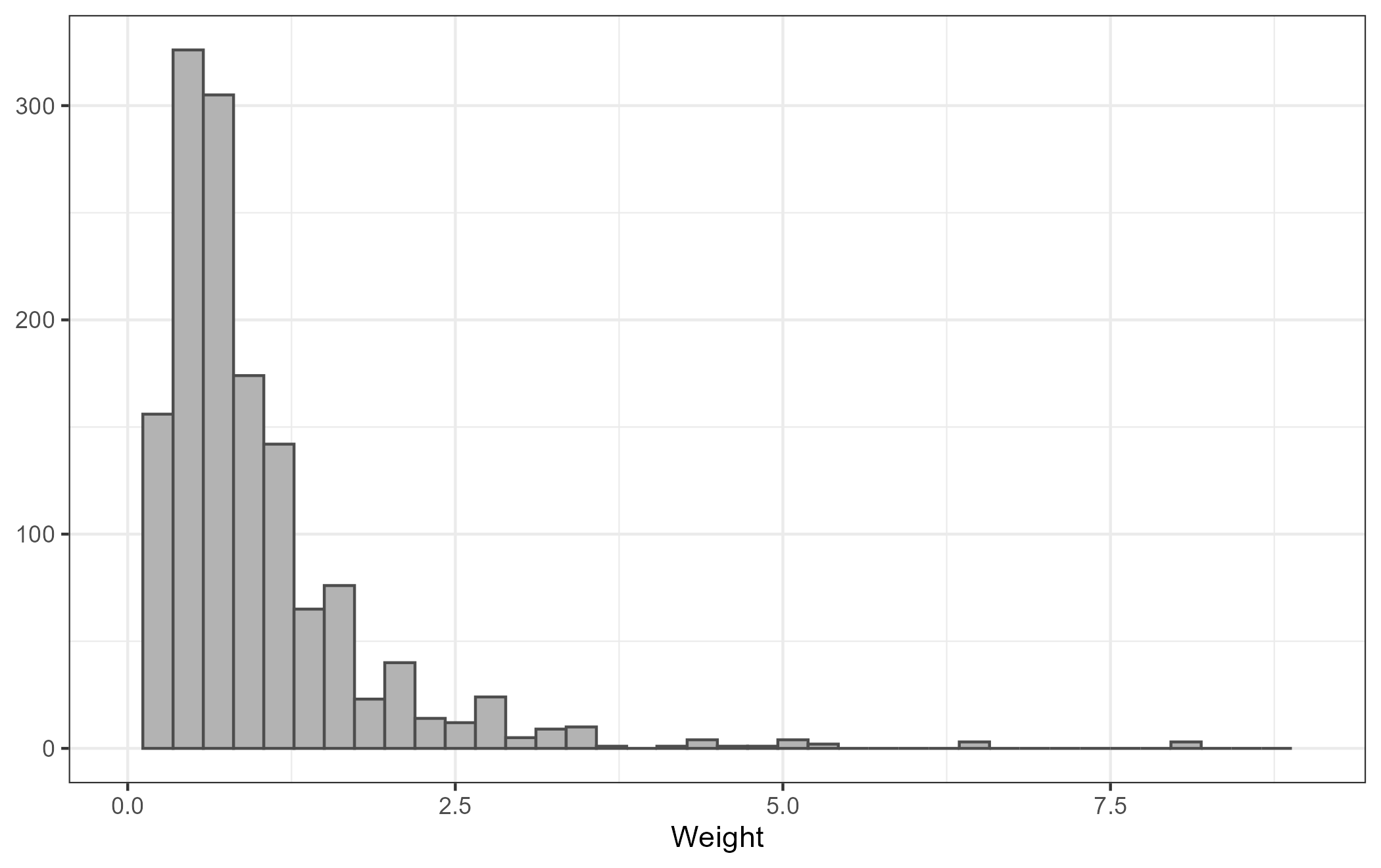


**Supplemental Table 1**. Characteristics and weights of survey respondents with 10 highest weights.

| Weight | Sex/gender | Age group | Race/ethnicity | Household size |
| --- | --- | --- | --- | --- |
| 8.19 | Male | 18-29 | Hispanic any race | Four or more |
| 8.19 | Male | 18-29 | Hispanic any race | Four or more |
| 8.19 | Non-binary/non-conforming | 18-29 | Hispanic any race | Four or more |
| 6.55 | Male | 18-29 | Hispanic any race | Four or more |
| 6.43 | Female | 18-29 | Hispanic any race | Four or more |
| 6.43 | Female | 18-29 | Hispanic any race | Four or more |
| 5.36 | Female | 18-29 | Hispanic any race | Four or more |
| 5.36 | Female | 18-29 | Hispanic any race | Four or more |
| 5.18 | Male | 18-29 | Asian alone | Four or more |
| 5.18 | Male | 18-29 | Asian alone | Four or more |
