# Supplementary material for: Exploring Attitudes and Obstacles Around Digital Public Health Tools: Insights From a Statewide Cross-Sectional Survey on Washington’s Vaccine Verification System
Source: J Med Internet Res. 2025 Oct 3;27:e66550. doi: 10.2196/66550 (PMC12534757; doi:10.2196/66550)
Supplement: Multimedia Appendix 3 [file jmir_v27i1e66550_app3.pdf]

STROBE Statement for **“Exploring Attitudes and Obstacles around Digital Public Health Tools: Insights from a Statewide Cross-Sectional Survey on Washington’s Vaccine Verification System”** — Checklist of items that should be included in reports of ***cross-sectional studies***

	Item No	Recommendation	Author’s Response
**Title and abstract**	1	(*a*) Indicate the study’s design with a commonly used term in the title or the abstract	Completed. Statewide, cross-sectional, and survey all used in to describe the study design in both the title and abstract
		(*b*) Provide in the abstract an informative and balanced summary of what was done and what was found	Completed. Background, objective, methods, results, and conclusions included in abstract
**Introduction**			
Background/rationale	2	Explain the scientific background and rationale for the investigation being reported	Completed. Background on the need and rollout of WA Verify technology during the COVID-19 pandemic, Technology Acceptance Model, and rationale for investigating these survey data included
Objectives	3	State specific objectives, including any prespecified hypotheses	Completed. Objective of the study included at end of Introduction
**Methods**			
Study design	4	Present key elements of study design early in the paper	Completed
Setting	5	Describe the setting, locations, and relevant dates, including periods of recruitment, exposure, follow-up, and data collection	Completed. Recruitment procedures and associated relevant dates included in Methods section and in Figure 2
Participants	6	(*a*) Give the eligibility criteria, and the sources and methods of selection of participants	Completed
Variables	7	Clearly define all outcomes, exposures, predictors, potential confounders, and effect modifiers. Give diagnostic criteria, if applicable	Completed. All variables used were clearly defined, including the question wording and response options
Data sources/ measurement	8*	For each variable of interest, give sources of data and details of methods of assessment (measurement). Describe comparability of assessment methods if there is more than one group	Completed. All data on respondents came directly from the statewide survey
Bias	9	Describe any efforts to address potential sources of bias	Completed. Post-stratification weights were created and implemented, as described in the Methods
Study size	10	Explain how the study size was arrived at	Completed. All respondents included in the analysis

			except for those with missing data on required user status questions, explained in Results
Quantitative variables	11	Explain how quantitative variables were handled in the analyses. If applicable, describe which groupings were chosen and why	Completed
Statistical methods	12	(*a*) Describe all statistical methods, including those used to control for confounding	Completed
		(*b*) Describe any methods used to examine subgroups and interactions	Completed
		(*c*) Explain how missing data were addressed	Completed. There was very little missing data for variables of interest (complete case analysis) and hot deck imputation used for post-stratification weights with missing demographic information
		(*d*) If applicable, describe analytical methods taking account of sampling strategy	Completed
		(*e*) Describe any sensitivity analyses	Completed. No sensitivity analyses completed, primarily a descriptive analysis
**Results**			
Participants	13*	(a) Report numbers of individuals at each stage of study—eg numbers potentially eligible, examined for eligibility, confirmed eligible, included in the study, completing follow-up, and analysed	Completed. 5000 individuals were invited to participate, 1491 completed the survey, and 1401 were included in the analysis. There was no longitudinal follow-up
		(b) Give reasons for non-participation at each stage	N/A
		(c) Consider use of a flow diagram	Did not complete
Descriptive data	14*	(a) Give characteristics of study participants (eg demographic, clinical, social) and information on exposures and potential confounders	Completed. Characteristics included in Table 1 with a comparison column for the state of Washington (source population)
		(b) Indicate number of participants with missing data for each variable of interest	Completed. Included in Table 1
Outcome data	15*	Report numbers of outcome events or summary measures	N/A, no formal outcome. All variables of interest presented in figures
Main results	16	(*a*) Give unadjusted estimates and, if applicable, confounder-adjusted estimates and their precision (eg, 95% confidence interval). Make clear which confounders were adjusted for and why they were included	N/A, all estimates were presented with the post-stratification weights. No adjusted analyses performed
		(*b*) Report category boundaries when continuous variables were categorized	N/A
		(*c*) If relevant, consider translating estimates of relative risk into absolute risk for a	N/A

		meaningful time period	
Other analyses	17	Report other analyses done—eg analyses of subgroups and interactions, and sensitivity analyses	Completed
**Discussion**			
Key results	18	Summarise key results with reference to study objectives	Completed. Key results summarized
Limitations	19	Discuss limitations of the study, taking into account sources of potential bias or imprecision. Discuss both direction and magnitude of any potential bias	Completed. Self-report nature and low survey response discussed as potential sources of bias, and insufficient sample size on non-white and non-Asian populations discussed as limitations
Interpretation	20	Give a cautious overall interpretation of results considering objectives, limitations, multiplicity of analyses, results from similar studies, and other relevant evidence	Completed. Results interpreted in the context of the WA Verify program and "vaccine passports" as a whole, the Technology Acceptance model, and related literature and work
Generalisability	21	Discuss the generalisability (external validity) of the study results	Completed. Discussed that results from this survey may not be applicable beyond Washington State
**Other information**			
Funding	22	Give the source of funding and the role of the funders for the present study and, if applicable, for the original study on which the present article is based	Completed

*Give information separately for exposed and unexposed groups.

Note: An Explanation and Elaboration article discusses each checklist item and gives methodological background and published examples of transparent reporting. The STROBE checklist is best used in conjunction with this article (freely available on the Web sites of PLoS Medicine at http://www.plosmedicine.org/, Annals of Internal Medicine at http://www.annals.org/, and Epidemiology at http://www.epidem.com/). Information on the STROBE Initiative is available at www.strobe-statement.org.
